# Intranasally Administered Extracellular Vesicles from Adipose Stem Cells Have Immunomodulatory Effects in a Mouse Model of Asthma

**DOI:** 10.1155/2021/6686625

**Published:** 2021-07-31

**Authors:** Sue Jean Mun, Shin Ae Kang, Hye-Kyung Park, Hak Sun Yu, Kyu-Sup Cho, Hwan-Jung Roh

**Affiliations:** ^1^Department of Otorhinolaryngology, Pusan National University Yangsan Hospital, Yangsan, Republic of Korea; ^2^Department of Parasitology and Tropical Medicine, Pusan National University School of Medicine, Yangsan, Republic of Korea; ^3^Department of Internal Medicine, Pusan National University Hospital, Busan, Republic of Korea; ^4^Department of Otorhinolaryngology, Pusan National University Hospital, Busan, Republic of Korea

## Abstract

Asthma is a chronic eosinophilic airway disease characterized by type 2 helper T cell-driven inflammation. Adipose stem cells (ASCs) and the ASC culture supernatant are known to improve allergic airway inflammation; however, the immunomodulatory effects of ASC-derived extracellular vesicles (EVs) on allergic airway diseases remain unclear. Thus, we assessed the effects of ASC-derived EVs on allergic airway inflammation in a mouse model of asthma. EVs were isolated from the culture supernatant of murine ASCs and characterized. Six-week-old female C57BL/6 mice were sensitized to ovalbumin (OVA) by intraperitoneal injection and challenged intranasally with OVA. Before the OVA challenge, 10 *μ*g/50 *μ*l of ASC-derived EVs was administered intranasally to the experimental group. ASC-derived EVs significantly attenuated airway hyperresponsiveness (AHR) in asthmatic mice (*p* = 0.023). ASC-derived EVs resulted in a remarkable reduction of the total number of inflammatory cells (*p* = 0.005) and eosinophils (*p* = 0.023) in the bronchoalveolar lavage fluid (BALF), the degree of eosinophilic lung inflammation (*p* < 0.001), and the serum total and OVA-specific immunoglobulin (Ig)E (*p* = 0.048 and *p* = 0.001) and total IgG1 (*p* < 0.001). Interleukin- (IL-) 4 was significantly inhibited with ASC-derived EV pretreatment in the BALF and lung draining lymph nodes (LLNs) (*p* = 0.040 and *p* = 0.011). Furthermore, ASC-derived EV administration resulted in a significant increase of the regulatory T cell (Treg) populations in LLNs. ASC-derived EVs alleviated AHR and allergic airway inflammation caused by the induction of Treg expansion in a mouse model of asthma. There seems to be a role for ASC-derived EVs as a modifier in allergic airway disease.

## 1. Introduction

Asthma is a chronic eosinophilic airway disease mainly driven by excessive activation of type 2 helper T (Th2) cells [1]. Th2 cell-driven inflammation is reflected by a significant increase in interleukin- (IL-) 4, IL-5, and IL-13 levels, causing airway eosinophilic inflammation, high serum immunoglobulin E (IgE) levels, and airway hyperresponsiveness (AHR) [2]. Regulatory T cells (Tregs), characterized by the expression of transcription factor Foxp3, are known to suppress allergic airway inflammation through surface molecules such as cytotoxic T lymphocyte-associated antigen 4, CD25, CD73, and CD39 [3–5].

Mesenchymal stem cells (MSCs) can regulate inflammation and immune responses [6]. Previous studies have shown that MSCs, including those derived from adipose tissue-derived stem cells (ASCs), can alleviate allergic airway inflammation in asthmatic mice [7–9]. ASC administration before the allergen challenge inhibited AHR, lung inflammation, and Th2 cytokine production through inhibition of Th2 cell activity in an asthmatic murine model [7]. Bone marrow-derived MSCs and human MSCs also inhibited Th2-mediated allergic airway inflammation in asthmatic mice [8, 9]. Although the complete immune-suppressive roles of MSCs are not understood, these cells are closely related to the induction of Treg expansion and increase in soluble factors, including IL-10 and transforming growth factor-*β* (TGF-*β*), resulting in the reduction of lung eosinophilic infiltration, Th2 cytokines, allergen-specific immunoglobulin production, and AHR [10–13].

Extracellular vesicles (EVs) are nanosized membranous vesicles. Various cell types secrete EVs into their surrounding extracellular space [14]. EVs contain proteins, mRNAs, and microRNAs (miRNAs) and exert their effects by delivering their contents to target cells [15, 16]. MSC-derived EVs can promote the regeneration of damaged tissues after myocardial infarction [17, 18], bone fracture [19], vascular injury [20], and cutaneous injury [21]. With respect to lung inflammation, MSC-derived EVs improved survival in a mouse model of *Escherichia coli* pneumonia infection with the same efficacy as that of MSCs [22]. However, the effects of MSC-derived EVs on allergic inflammation in asthmatic mice remain unclear.

In this study, EVs isolated from the culture supernatant of murine ASCs (ASC sup) were used because of their abundance, relatively easy harvesting, and high proliferation potential [23]. The effects of ASC-derived EVs on allergic airway inflammation, type 1 helper T- (Th1-), Th2-, and Treg-related cytokine levels, and Treg induction were investigated in an ovalbumin- (OVA-) induced asthmatic mouse model.

## 2. Materials and Methods

### 2.1. Animals

Six-week-old female C57BL/6 mice (Samtako Co., Osan, Republic of Korea) were bred in pathogen-free animal facilities. This study protocol was approved by the Institutional Animal Care and Use Committee of the Pusan National University School of Medicine (Approval No. PNU-2016-1109).

### 2.2. EV Extraction and Characterization

As in our previous studies [11, 23–25], adipose tissue was harvested from the abdominal fat of C57BL/6 mice. ASCs were cultured (37°C, 5% CO_2_) in *α*-modified Eagle's medium (*α*-MEM) containing 10% fetal bovine serum (FBS) until they reached 1 × 10^6^ cells/cm^2^. EVs were isolated from the ASC sup [26]. The ASC sup was filtered through a 0.45 *μ*m vacuum filter. A QuixStand (GE Healthcare, Little Chalfont, UK) and 0.22 *μ*m bottle top filter (Sigma-Aldrich, St. Louis, MO, USA) were used for the concentration and filtration processes. The filtrates were pelleted by ultracentrifugation in a 45 Ti rotor (Beckman Coulter, Fullerton, CA, USA) at 100,000 g for 2 hours at 4°C. Then, the resulting pellets were resuspended in PBS and stored at -80°C. The EVs were then placed on 300-mesh copper grids and stained with 2% uranyl acetate. Approximately 4-5 × 10^7^ ASCs were used to obtain 10 *μ*g of EVs.

A JEM-1011 transmission electron microscope (TEM) (JEOL, Tokyo, Japan) operated at 100 kV, Zetasizer Nano ZS (Malvern Instruments Ltd., Malvern, UK) with a 633 nm laser line, at a scatter intensity of 10 × 30 s, and NanoSight LM10 (Malvern Instruments Ltd.) with NanoSight nanoparticle tracking analysis (NTA) 2.3 software were used to characterize EVs [27, 28].

CD81 and CD40, which are markers of EVs, and calnexin, which is an endoplasmic reticulum protein associated with compartments other than plasma membranes or endosomes, were analyzed by western blotting with the primary antibodies, anti-CD81 (1 : 1000, Abcam, Cambridge, UK), anti-CD40 (1 : 1000, Abcam), and anti-calnexin (1 : 1000, GeneTex, Irvine, CA) [29]. To analyze CD81 and CD40 expression, 30 *μ*g of EV was separated by Mini-PROTEAN TGX Gels (Bio-Rad, Hercules, CA, USA) electrophoresis at 100 V for 90 minutes. The separated proteins were transferred onto polyvinylidene difluoride membranes (Amersham Biosciences, Amersham, UK), and the membranes were blocked overnight with 5% skim milk in Tris-buffered saline containing 0.1% Triton X-100. The membranes were washed five times and incubated with anti-CD81 (rabbit monoclonal Ab, # D502Q) (Cell Signaling Technology, Danvers, MA, USA), anti-CD40 (rabbit polyclonal Ab, ADI-CSS-180) (Enzo Life Sciences, Farmingdale, NY, USA), and anti-calnexin (rabbit polyclonal Ab, # GTX112886) (GeneTex, Irvine, CA, USA), diluted at 1 : 1000 in the blocking buffer, overnight at 4°C. Detection was performed with horseradish peroxidase-conjugated secondary anti-rabbit antibodies used at a 1 : 1000 dilution for 1 hour at room temperature. The blot for horseradish peroxidase was developed using the ECL substrate (Amersham Biosciences).

### 2.3. Mouse Model of Allergic Airway Inflammation

**Figure 1:**
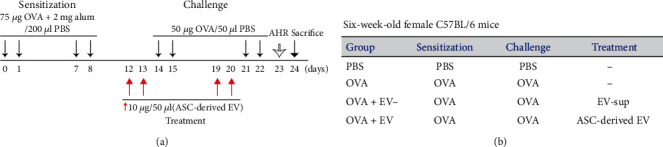
The experimental protocol. (a) Mice were sensitized on days 0, 1, 7, and 8 by intraperitoneal injection of ovalbumin (OVA) and then challenged intranasally with OVA on days 14, 15, 21, and 22. On days 12, 13, 19, and 20, 10 *μ*g/50 *μ*l of the adipose stem cell- (ASC-) derived extracellular vesicles (EVs) was administered intranasally. (b) Mice were divided into 4 groups according to different sensitization, challenge, and pretreatment methods.

A mouse model of asthma was employed with some modifications from previous studies [23–25]. The mice were sensitized by intraperitoneal injection with 75 *μ*g of OVA (Sigma-Aldrich) with 2 mg of aluminum hydroxide (Sigma-Aldrich) in 200 *μ*l PBS on days 0, 1, 7, and 8. The mice were then challenged intranasally with 50 *μ*g OVA in 50 *μ*l PBS on days 14, 15, 21, and 22. The mice were evaluated for lung function on day 23 and sacrificed on day 24 (Figure 1(a)).

### 2.4. Intranasal Administration of ASC-Derived EVs

To assess the effects of ASC-derived EVs, 10 *μ*g/50 *μ*l of EVs was administered intranasally on days 12, 13, 19, and 20. The mice were divided into four groups, with five mice per group: (a) PBS group: sensitized, pretreated, and challenged with PBS; (b) OVA group: sensitized with OVA, pretreated with PBS, and then challenged with OVA; (c) OVA+EV- group: sensitized with OVA, pretreated with ASC sup without EV, and then challenged with OVA; and (d) OVA+EV group: sensitized with OVA, pretreated with ASC-derived EVs, and then challenged with OVA (Figure 1(b)).

### 2.5. Measurement of AHR

As described in previous studies, AHR was evaluated in awake mice by noninvasive whole-body plethysmography (Allmedicus, Seoul, Republic of Korea) after 24 hours from the last challenge [11, 23, 24, 30]. The mice were exposed to aerosolized methacholine for 10 min at 0, 12.5, 25, and 50 mg/ml. The enhanced pause (Penh) was automatically measured according to the mean pressure generated in the chamber during inspiration and expiration combined with the time. The mean Penh values calculated during each 3 min term were shown.

### 2.6. Differential Cell Counting in Bronchoalveolar Lavage Fluid

The bronchoalveolar lavage fluid (BALF) was collected after sacrificing the mice as described in our previous studies [11, 23, 24, 30]. The samples were centrifuged at 1500 rpm for 5 min at 4°C. Then, the supernatants of the BALF were immediately frozen at -70°C. Cell pellets were resuspended and washed in PBS. The total cell counts were evaluated using a hematocytometer. BALF cell smears were stained with Diff-Quik solution (Sysmex Co., Kobe, Japan) to identify the differential cell counts according to the conventional morphological criteria. To evaluate the differential leukocyte counts, a minimum of 500 cells per slide were examined.

### 2.7. Lung Histology and Inflammation Score

After lavage, the lung tissue was removed, fixed in 10% neutral formalin for 36 hours, and then embedded in paraffin. To identify eosinophils, the sections were stained with hematoxylin and eosin (H&E). The index of lung inflammation was assessed by scoring the inflammatory cell infiltrates around vessels and airways for the greatest severity (0, normal; 1, ≤3 cells thick; 2, 4-10 cells thick; and 3, ≥10 cells thick) and overall extent (0, normal; 1, <25% of the sample; 2, 25-50% of the sample; 3, 51-75% of the sample; and 4, ≥75% of the sample) [30]. The score was calculated by multiplying the severity by the extent.

### 2.8. Measurement of Serum Immunoglobulin

Forty-eight hours after the last challenge, the serum was prepared from blood obtained via cardiac puncture of mice. Total and OVA-specific immunoglobulins (IgE, IgG1, and IgG2a) were evaluated by enzyme-linked immunosorbent assay (ELISA) according to the manufacturer's instructions (R&D Systems, Minneapolis, MN, USA). Absorbance at 450 nm was determined using an ELISA plate reader (Molecular Devices, Sunnyvale, CA, USA).

### 2.9. Analysis of Cytokine Expression in the BALF and Lung Draining Lymph Nodes

After sacrifice, lung draining lymph nodes (LLNs) were harvested in between the trachea and bilateral lung lobes of mice. The lymphocytes were isolated from the LLNs as previously reported [11, 23, 24, 31] and plated in 48-well plates coated with a 0.5 *μ*g/ml CD3 antibody (BD Pharmingen™, BD Biosciences, San Jose, CA, USA) at a concentration of 10^6^ cells/ml in RPMI 1640 with 10% FBS. Cells were incubated for 72 hours at 37°C with 5% CO_2_. The supernatant of LLNs was used after stimulation. The concentrations of IL-4, IL-5, IL-13, and interferon- (IFN-) *γ* in the BALF and in the LLN supernatants were determined by ELISA kits following the manufacturer's recommendations (eBioscience, San Diego, CA, USA). The absorbance of the final reactant was measured at 450 nm using an ELISA plate reader.

### 2.10. FACS Analysis of T Cell Populations in LLNs

To assess the recruitment of Th1, Th2, and Tregs induced by treatment with ASC-derived EVs, LLN cells from mice in the asthmatic and experimental groups were cultured in anti-CD3-coated plates for 6 hours. To determine the CD4^+^CD25^+^Foxp3^+^ Treg cell populations, the cells were stained with anti-CD4-FITC (0.5 mg/ml) and anti-CD25-APC (0.2 mg/ml) according to the manufacturer's instructions (eBioscience). The cells were then fixed with a Cytofix/Cytoperm kit (eBioscience), permeabilized, and incubated with anti-Foxp3-PE-cy7 (eBioscience).

To sort the Th1 and Th2 cell populations, LLN cells were stained with the anti-CD4-FITC antibody. The CD4^+^ T cells were then stained with intracellular anti-IFN-*γ*-PE-cy7 (eBioscience) and anti-IL-4-PE (eBioscience) antibodies. Fluorescence was determined using a FACSCanto II cytometer (BD Biosciences) equipped with Canto software (BD Biosciences).

### 2.11. Statistical Analysis

Each independent experiment was performed at least three times. Data are represented as the mean ± standard error of the mean. Statistical significance was assessed by one-way ANOVA with the Bonferroni *post hoc* test or Kruskal-Wallis test followed by Dunn's posttest using GraphPad Prism 5.0 software (GraphPad Software Inc., La Jolla, CA). A value of *p* < 0.05 was considered significant.

## 3. Results

### 3.1. Characterization of ASC-Derived EVs

**Figure 2:**
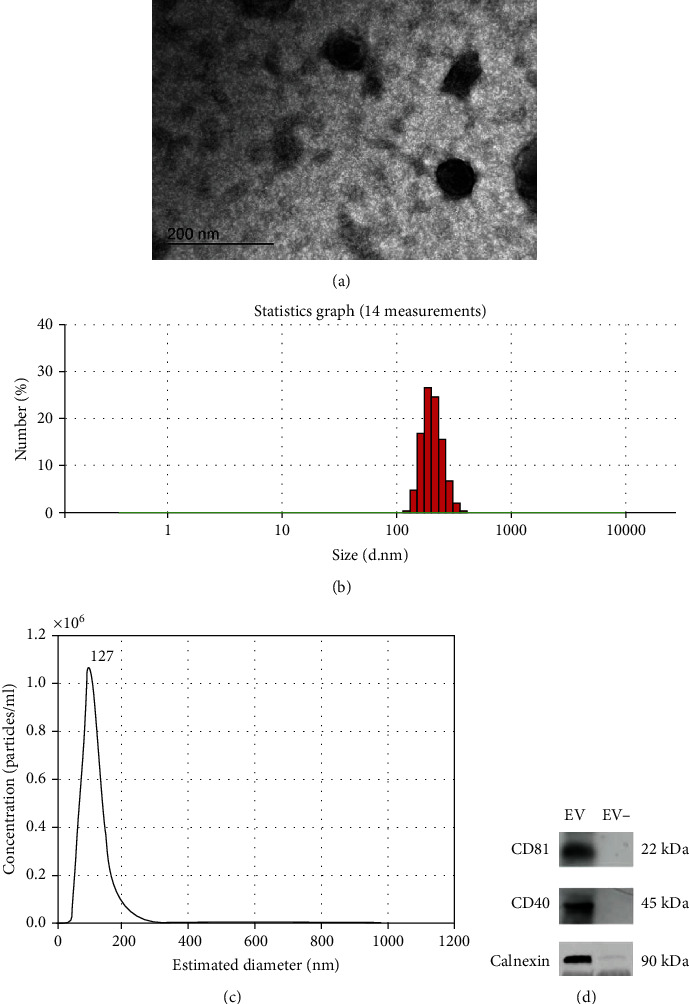
Characterization of adipose stem cell- (ASC-) derived extracellular vesicles (EVs). (a) Transmission electron microscopy of ASC-derived EVs showed a spherical shape and lipid bilayers (original magnification ×250,000; the scale bar indicates 200 nm). (b) Average size of the EVs calculated by dynamic light scattering was 211.7 ± 26.6 nm, and average concentration was 57,946,000 ± 12,519,069 particles/ml. (c) Nanoparticle tracking analysis revealed corresponding EV size distribution and a mode of 127.1 ± 3.2 nm. (d) Western blotting of EVs showed high expression of CD81, CD40, and calnexin.

TEM showed that ASC-derived EVs were composed of lipid bilayers and were spherical in shape (Figure 2(a)). The average size of the EVs as determined by dynamic light scattering (DLS) was 211.7 ± 26.6 nm (Figure 2(b)). The majority of particle sizes were in the range of 100-400 nm. The NTA revealed a mode of 127.1 ± 3.2 nm, which was in accordance with the results of TEM and DLS (Figure 2(c)). Western blotting analysis revealed high levels of both CD81 and CD40, which are known as EV-enriched markers, and calnexin, which is an endoplasmic reticulum protein (Figure 2(d)).

### 3.2. AHR and Differential Cell Counting in BALF

**Figure 3:**
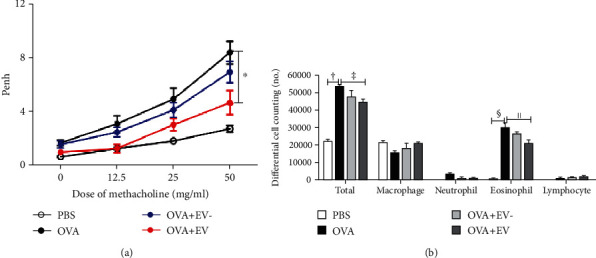
Effects of adipose stem cell- (ASC-) derived extracellular vesicles (EVs) on airway hyperresponsiveness (AHR) and differential cell counts in bronchoalveolar lavage fluid (BALF). (a) After the methacholine challenge, AHR decreased significantly in the OVA+EV group compared to the OVA group. (b) Total inflammatory cell and eosinophil counts in BALF significantly lowered in the OVA+EV group compared to the OVA group. Data are expressed as the mean ± SEM of four independent experiments, each performed in triplicate. ^∗^*p* = 0.023, ^†,§^*p* < 0.001, and ^‡^*p* = 0.005.

The Penh values were increased with the methacholine concentration in asthmatic mice. Treatment with ASC-derived EVs significantly decreased AHR in asthmatic mice (*p* = 0.023). However, ASC sup treatment without EV did not significantly influence AHR (Figure 3(a)).

The total inflammatory cell and eosinophil counts were markedly increased in the BALF of the OVA group compared to the PBS group. However, pretreatment with ASC-derived EVs remarkably lowered the total inflammatory cell (*p* = 0.005) and eosinophil (*p* = 0.023) counts in asthmatic mice. However, ASC sup treatment without EV did not reduce BALF total inflammatory cell or eosinophil counts in asthmatic mice (Figure 3(b)).

### 3.3. Lung Histology and Inflammation Score

**Figure 4:**
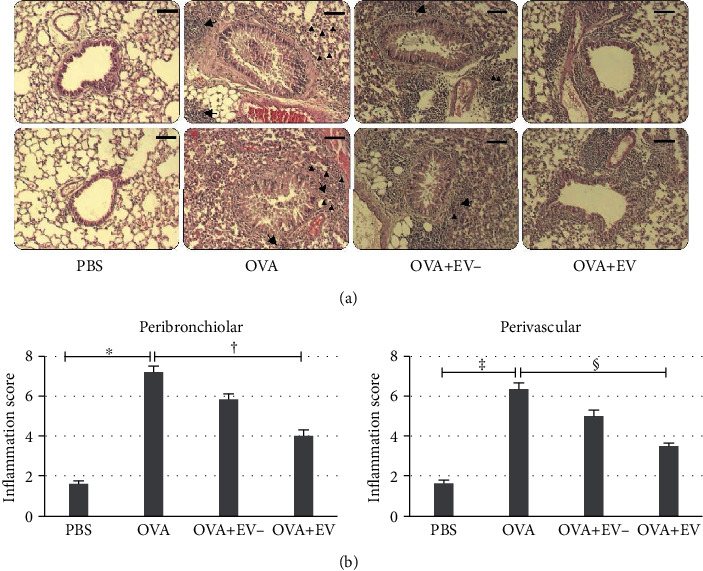
Effects of adipose stem cell- (ASC-) derived extracellular vesicles (EVs) on lung histology and inflammation scores. (a) Infiltration of inflammatory cells (black arrow) and eosinophils (black arrowhead) showed a greater decrease in the OVA+EV group than in the OVA group. The scale bar indicates 100 *μ*m. (b) The inflammation score decreased significantly in the OVA+EV group compared with the OVA group around peribronchiolar and perivascular areas. Data are expressed as the mean ± SEM of four independent experiments, each performed in triplicate. ^∗^^,†,‡,§^*p* < 0.001.

High levels of eosinophil infiltration were observed around the peribronchiolar and perivascular areas in asthmatic mice. However, in the group treated with ASC-derived EVs, there was no obvious infiltration of inflammatory cells around these areas (Figure 4(a)). Furthermore, the inflammation score was markedly decreased in the ASC-derived EV-treated group compared to the OVA group (all *p* < 0.001) (Figure 4(b)).

### 3.4. Serum Total and OVA-Specific IgE, IgG1, and IgG2a Levels

**Figure 5:**
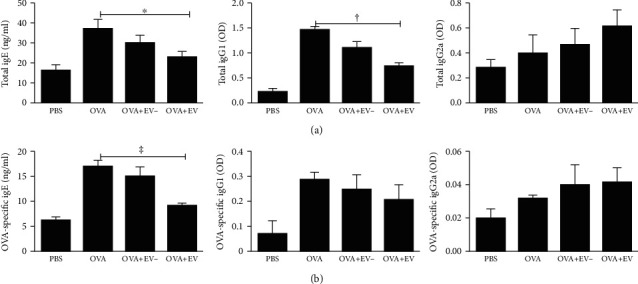
Effects of adipose stem cell- (ASC-) derived extracellular vesicles (EVs) on serum immunoglobulin levels by ELISA. Total (a) and OVA-specific (b) IgE and IgG1 levels were markedly raised in the OVA group compared to the PBS group. Intranasal administration of ASC-derived EVs significantly reduced the total (a) and OVA-specific (b) IgE and IgG1 levels in asthmatic mice. Data are expressed as the mean ± SEM of four independent experiments, each performed in triplicate. ^∗^*p* = 0.011, ^†^*p* < 0.001, and ^‡^*p* = 0.001.

The total and OVA-specific IgE and IgG1 levels were remarkably increased in the OVA group compared to the PBS group. However, intranasal administration of ASC-derived EVs significantly lowered the total IgE and IgG1 (*p* = 0.048 and *p* < 0.001, respectively), as well as OVA-specific IgE (*p* = 0.001) in asthmatic mice. No significant changes were observed in the total and OVA-specific IgG2a levels in all groups (Figure 5).

### 3.5. Expression of Cytokines in the BALF and LLNs

**Figure 6:**
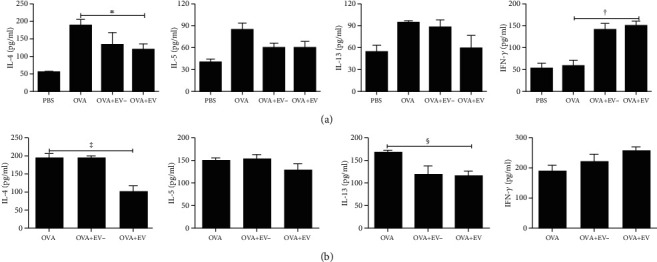
Effects of adipose stem cell- (ASC-) derived extracellular vesicles (EVs) on cytokine levels by ELISA in the bronchoalveolar lavage fluid (BALF) (a) and lung draining lymph nodes (LLNs) (b). IL-4, IL-5, and IL-13 levels were significantly increased in the BALF of the OVA group compared to the PBS group. Treatment with ASC-derived EVs remarkably reduced the levels of IL-4 but increased the levels of IFN-*γ* in the BALF. ASC-derived EV treatment notably decreased the levels of IL-4 and IL-13 in the LLNs. Data are expressed as the mean ± SEM of four independent experiments, each performed in triplicate. ^∗^*p* = 0.040, ^†^*p* = 0.006, ^‡^*p* = 0.011, and ^§^*p* = 0.012.

OVA-induced asthmatic mice showed a significant increase in the levels of IL-4, IL-5, and IL-13 in their BALF. However, treatment with ASC-derived EVs markedly lowered the IL-4 level in the BALF of asthmatic mice (*p* = 0.040). In contrast, ASC-derived EV treatment remarkably increased the IFN-*γ* level in the BALF in asthmatic mice (*p* = 0.006). Similarly, LLNs with intranasally administered ASC-derived EVs before the OVA challenge showed considerable reductions in the levels of IL-4 and IL-13 in the asthmatic mice (*p* = 0.011 and *p* = 0.012, respectively) (Figure 6).

### 3.6. T Cell Populations in the LLNs

**Figure 7:**
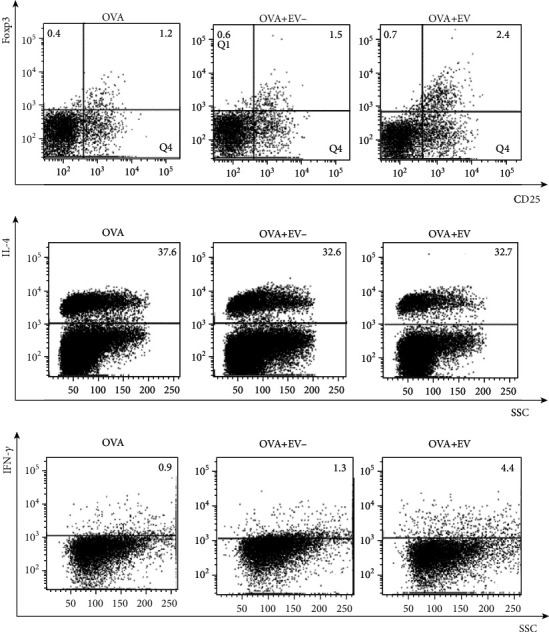
Effects of adipose stem cell- (ASC-) derived extracellular vesicles (EVs) on T cell populations in the lung draining lymph nodes. The populations of CD4^+^CD25^+^Foxp3^+^ T cells were markedly increased by administration of ASC-derived EVs in the OVA group. ASC-derived EV administration significantly reduced CD4^+^IL-4^+^ T cells but promoted CD4^+^IFN-*γ*^+^ T cells.

Intranasally administered ASC-derived EVs notably increased the populations of CD4^+^CD25^+^Foxp3^+^ T cells in asthmatic mice compared to the OVA group and the group pretreated with ASC sup without EV. ASC-derived EVs significantly decreased the CD4^+^IL-4^+^ T cell population and considerably increased the CD4^+^IFN-*γ*^+^ T cell population compared to the OVA group and the group pretreated with ASC sup without EV (Figure 7).

## 4. Discussion

MSCs have been reported as promising candidates to treat allergic airway diseases, as they can modulate immune function. Although the effects of MSCs may be triggered by direct contact between MSCs and CD4^+^ T cells, the main protective effects are predominantly paracrine mediated [32, 33]. MSCs secrete a variety of autocrine and paracrine factors including cytokines, chemokines, and growth factors that provide similar therapeutic effects as systemically administered MSCs [31, 34, 35]. ASC sup-containing EVs reverse the course of allergic airway inflammation by suppressing Th2 cytokine release and induction of Treg expansion even without ASCs [29, 31]. Furthermore, compared with ASC transplantation, cell-free therapies mediated by ASC sup or ASC-derived EVs have many advantages including safety, ease of handling or storage, low possibility of immune rejection, and no risk of aneuploidy or vascular occlusion [36].

EVs are released by most of the cell types under physiological or pathological conditions. They represent endogenous cargo capable of participating in cell-to-cell communication [33, 37]. Systemically administered human bone marrow-derived MSC-derived EVs were shown to ameliorate *Aspergillus* hyphal extract-induced eosinophilic and neutrophilic airway inflammation in experimental mice [38]. Additionally, MSC-derived EVs upregulated TGF-*β*1 and IL-10 in the peripheral blood mononuclear cells of asthmatic patients and promoted the proliferation and immune-suppressive capacity of Tregs [39]. Furthermore, ASC-derived EVs ameliorated *Aspergillus* protease antigen-induced Th2-mediated inflammation by activating dendritic cells and inducing M2 macrophage polarization. Thus, ASC-derived EVs downregulated IL-25 and eotaxin and upregulated IL-10 and TGF-*β* in mouse lung epithelial cells [29]. Considering the growing body of evidence suggesting that the paracrine effects mediated by EVs modulate immune function, the plausible roles of ASC-derived EVs in a mouse allergic airway model were investigated in this study.

Recent consensus guidelines have endorsed the nomenclature of EVs as a generic term for particles naturally released from cells. Typically, EVs with diameters of 40-100 nm that originate from multivesicular endosomes are considered exosomes. In contrast, EVs with diameters of 100-1000 nm and which bud from the cell surface are designated as microvesicles [14, 40, 41]. Isolated EVs are characterized by a combination of methods such as single vesicle analysis and determination of their protein composition. Single vesicle analysis involves electron microscopy or atomic force microscopy, single particle analyzers such as DLS and NTA, and high-resolution flow cytometry. EV proteins, including different surface markers such as tetraspanin CD63 and/or CD81, are identified by western blotting or mass spectrometry [28, 41, 42]. In this study, ASC-derived EVs were characterized by their spherical bodies using TEM. Their size ranged from 100 nm to 400 nm by DLS, and a mixture of exosomes and microvesicles was revealed by NTA. Furthermore, western blotting analysis showed high levels of the exosome-enriched marker, CD81, and the microvesicle-enriched marker, CD40, which agreed with the results of single vesicle analysis.

The present study showed that intranasal administration of ASC-derived EVs to asthmatic mice resulted in remarkable attenuation of allergic airway inflammation and marked alleviation of AHR. ASC-derived EVs significantly reduced the total inflammatory cells and eosinophils in the BALF and improved lung pathology. These findings suggest that ASC-derived EVs suppressed eosinophil recruitment to the lung and BALF and decreased AHR in asthmatic mice, which is consistent with the results of previous studies using MSCs or the MSC secretome [7, 11–13, 33]. Although ASC sup treatment without EV has a positive effect on lung histology and inflammation scores compared to the OVA group, there was no significant difference between the OVA+EV- group and the OVA group. This suggests that ASC-derived EVs play a critical role in the suppression of allergic airway inflammation, but the soluble proteins within the ASC sup are responsible for immunomodulatory effects in asthmatic mice. Our results were contrary to another study [43], using human ASCs and ASC-derived EVs in an allergic asthma murine model. Although our previous and present studies showed improved AHR and decreased eosinophils in the lung parenchyma in both the ASC sup and ASC-derived EVs, only ASC-derived EVs showed a significant reduction of static lung elastance and eosinophils in the lung parenchyma in this study. The important difference was the experimental protocol, as ASC sup and ASC-derived EVs were pretreated in our study and ASCs and ASC-derived EVs were treated after the challenge in the other study, which means after airway remodeling was already established. Another study [44] showed greater benefit in reducing levels of fibrogenesis-related growth factors in bone marrow-derived mononuclear cells than MSCs in an allergic asthma murine model, which might be further studied in the future.

Treatment with ASC-derived EVs significantly lowered the serum total and OVA-specific IgE levels and the total IgG1 levels. The level of IL-4 was significantly decreased in the BALF and LLNs, whereas IFN-*γ* was significantly increased in the BALF. Although the reason for the lack of a significant decrease in IL-5 level is unclear, our study demonstrated that ASC-derived EVs significantly alleviated Th2 response in asthmatic mice. Additionally, CD4^+^IL-4^+^ T cells were markedly decreased after ASC-derived EV treatment, whereas CD4^+^IFN-*γ*^+^ T cells were notably increased in the LLNs. Together with the attenuation of allergic airway inflammation by MSCs or the MSC secretome, these findings indicate that Treg expansion plays a key role in the immunomodulatory properties of ASC-derived EVs. Although the fundamental mechanism underlying the suppression of Th2-mediated inflammation in ASC-derived EVs remains unclear, our results indicate that EVs function as cargo containing mRNAs, regulatory miRNAs, cytokines, signaling lipids, and proteins [16] and may affect Tregs via various mechanisms. In EVs, these contents are known to be protected from enzymatic degradation. In particular, miRNAs, also known as exosomal shuttle RNAs [16], are endogenous, single-strand, noncoding RNAs that regulate translation by interacting with mRNA transcripts. Some miRNAs have been suggested to affect IL-13 and Th2 response, which are known to be fine-tuners in the mechanism of asthma [45].

Our study had some limitations. We were unable to distinguish whether the immunomodulatory effects of ASC-derived EVs were mediated by exosomes, microvesicles, or a combination of these. Further studies are needed to identify the EV contents accounting for the suppression of allergic airway inflammation in asthmatic mice. Molecular and genetic research including microarray DNA hybridization may also be required to elucidate the immune-suppressive mechanism of ASC-derived EVs in allergic airway inflammation. Furthermore, human experiments provide more biological complexity than murine model experiments. Therefore, the plausible role of ASC-derived EVs in allergic airway inflammation should be evaluated in human models.

## 5. Conclusions

Intranasally administered ASC-derived EVs significantly decreased allergic airway inflammation and improved AHR through induction of Treg expansion in asthmatic mice. Thus, ASC-derived EVs may act as a modifier in allergic airway disease such as asthma.

## Data Availability

The supporting data for this publication are available upon request.

## Abbreviations

ASCs: Adipose stem cells
ASC sup: ASC supernatant
BALF: Bronchoalveolar lavage fluid
DLS: Dynamic light scattering
ELISA: Enzyme-linked immunosorbent assay
EVs: Extracellular vesicles
FBS: Fetal bovine serum
H&E: Hematoxylin and eosin
IFN: Interferon
Ig: Immunoglobulin
IL: Interleukin
LLNs: Lung draining lymph nodes
LPS: Lipopolysaccharide
miRNAs: MicroRNAs
MEM: Modified Eagle's medium
MSCs: Mesenchymal stem cells
NTA: Nanoparticle tracking analysis
OVA: Ovalbumin
PBS: Phosphate-buffered saline
PCR: Polymerase chain reaction
Penh: Enhanced pause
PGE2: Prostaglandin E2
TEM: Transmission electron microscopy
TGF: Transforming growth factor
Tregs: Regulatory T cells.
